# 1-(4-*tert*-Butyl­benz­yl)-2-(4-*tert*-butyl­phen­yl)-1*H*-benzimidazole

**DOI:** 10.1107/S1600536809045668

**Published:** 2009-11-07

**Authors:** Jian-Cheng Zhou, Zheng-Yun Zhang, Nai-Xu Li, Chuan-Ming Zhang

**Affiliations:** a College of Chemistry and Chemical Engineering, Southeast University, Nanjing 211189, People’s Republic of China; b Jiangsu Provincial Key Laboratory of Pulp and Paper Science and Technology, Nanjing Forestry University, Nanjing 210037, People’s Republic of China

## Abstract

In the mol­ecule of the title compound, C_28_H_32_N_2_, the benzimidazole ring system is almost planar [maximum deviation = 0.0221 (15) Å] and forms dihedral angles of 85.86 (4) and 32.09 (6)° with the benzene rings. In the crystal structure, mol­ecules are linked into chains running parallel to the *a* axis by inter­molecular C—H⋯N hydrogen bonds. The methyl groups of a *tert*-butyl group are rotationally disordered over two positions with refined site-occupancy factors of 0.636 (4) and 0.364 (4).

## Related literature

For the biological and pharmaceutical properties of benzimidazole derivatives, see: Matsuno *et al.* (2000 ▶). Garuti *et al.* (1999 ▶). For reference structural data, see: Allen *et al.* (1987 ▶).
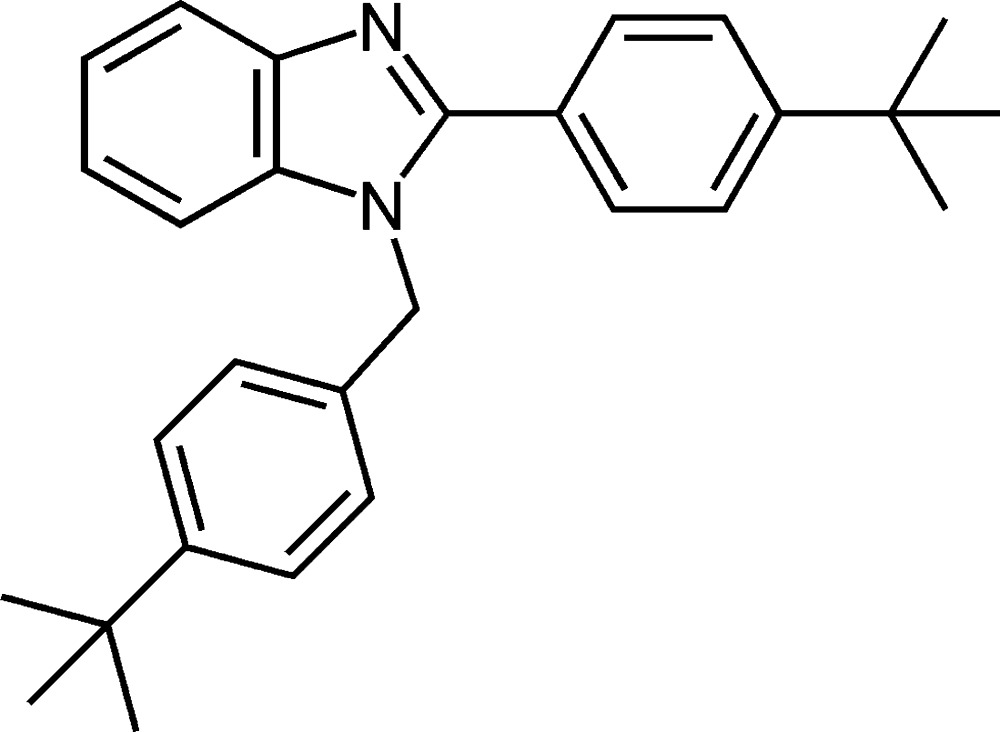


## Experimental

### 

#### Crystal data

C_28_H_32_N_2_
*M* *_r_* = 396.56
Monoclinic, 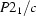

*a* = 6.2142 (5) Å
*b* = 21.1112 (13) Å
*c* = 17.4624 (12) Å
β = 92.869 (6)°
*V* = 2288.0 (3) Å^3^
*Z* = 4
Mo *K*α radiation
μ = 0.07 mm^−1^
*T* = 293 K
0.20 × 0.20 × 0.10 mm

#### Data collection

Rigaku SCXmini diffractometer
Absorption correction: multi-scan (*CrystalClear*; Rigaku, 2005 ▶) *T*_min_ = 0.987, *T*_max_ = 0.993
24819 measured reflections
5239 independent reflections
4315 reflections with *I* > 2σ(*I*)
*R*_int_ = 0.047

#### Refinement

*R*[*F* ^2^ > 2σ(*F* ^2^)] = 0.061
*wR*(*F* ^2^) = 0.135
*S* = 1.09
5239 reflections
302 parameters
H-atom parameters constrained
Δρ_max_ = 0.22 e Å^−3^
Δρ_min_ = −0.18 e Å^−3^

### 

Data collection: *CrystalClear* (Rigaku, 2005 ▶); cell refinement: *CrystalClear*; data reduction: *CrystalClear*; program(s) used to solve structure: *SHELXS97* (Sheldrick, 2008 ▶); program(s) used to refine structure: *SHELXL97* (Sheldrick, 2008 ▶); molecular graphics: *SHELXTL* (Sheldrick, 2008 ▶); software used to prepare material for publication: *SHELXL97*.

## Supplementary Material

Crystal structure: contains datablocks I, global. DOI: 10.1107/S1600536809045668/rz2378sup1.cif

Structure factors: contains datablocks I. DOI: 10.1107/S1600536809045668/rz2378Isup2.hkl

Additional supplementary materials:  crystallographic information; 3D view; checkCIF report

## Figures and Tables

**Table 1:** Hydrogen-bond geometry (Å, °)

*D*—H⋯*A*	*D*—H	H⋯*A*	*D*⋯*A*	*D*—H⋯*A*
C18—H18*A*⋯N2^i^	0.97	2.59	3.553 (2)	174

Symmetry code: (i) 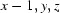.

**Fig. 1.**
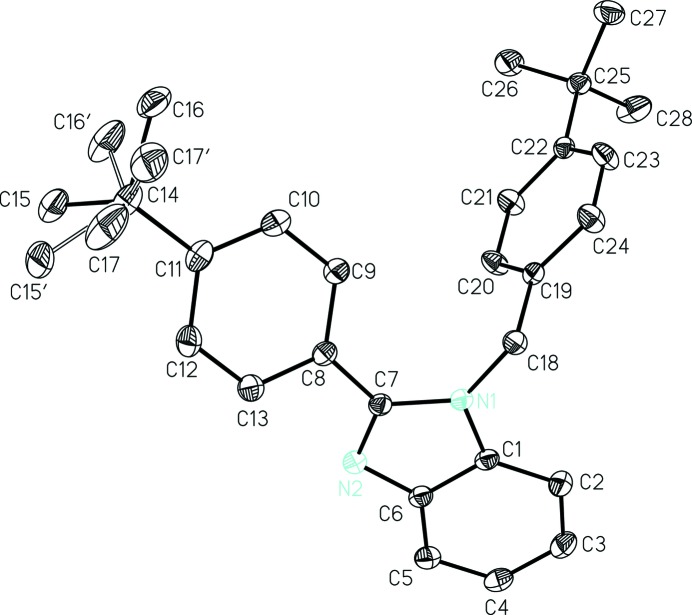
The molecular structure of the title compound, showing the atomic numbering scheme. The displacement ellipsoids are drawn at the 30% probability level.

## supplementary crystallographic information

### Comment

Imidazole and benzimidazole derivatives are important heteroaromatic compounds which have attracted great attention due to their biological and pharmaceutical activities (Matsuno *et al.*, 2000; Garuti *et al.*, 1999). These compounds have also played an important role in the development of coordination chemistry. In this paper, we report the crystal structure of the title compound.

The molecular structure of the title compound is shown in Fig. 1. Bond lengths (Allen *et al.*, 1987) and angles are within normal ranges. The benzimidazole ring system is substantially planar, with a maximum displacement of 0.0221 (15) Å for atom C1. The dihedral angle it forms with the C8—C13 and C19—C24 benzene rings are 32.09 (6) and 85.86 (4) Å, respectively. The benzene rings are oriented perpendicularly to each other, forming a dihedral angle of 89.58 (5) °. In the crystal packing, the molecule are linked into chains running parallel to the *a* axis by intermolecular C—H···N hydrogen bonds (Table 1). The methyl groups of a *tert*-butyl group exhibits rotational disorder over two orientations with site occupation factors of 0.636 (4) and 0.364 (4) for the major and minor components of disorder, respectively.

### Experimental

To a solution of *o*-phenylenediamine (0.432 g, 4 mmol) in ethanol(20 ml), 4-*tert*-butylbenzaldehyde (1.297 g, 8 mmol) was added. The mixture was heated to reflux with stirring for four hour, then cooled to room temperature. The resultant solution was filtered and allowed to evaporate slowly at room temperature. Colourless single crystals of the title compound suitable for X-ray diffraction study were obtained after several weeks.

### Refinement

The C15, C16 and C17 methyl carbon atoms of a *tert*-butyl group are rotationally disordered over two position with refined site occupancy factors of 0.636 (4) and 0.364 (4). All H atoms were located geometrically and treated as riding atoms, with C—H = 0.93–0.97 Å, and with *U*_iso_(H) = 1.2 *U*_eq_(C) or 1.5 *U*_eq_(C) for methyl H atoms.

### Crystal data

**Table:**

C_28_H_32_N_2_	*F*(000) = 856
*M**_r_* = 396.56	*D*_x_ = 1.151 Mg m^−^^3^
Monoclinic, *P*2_1_/*c*	Mo *K*α radiation, λ = 0.71073 Å
Hall symbol: -P 2ybc	Cell parameters from 3426 reflections
*a* = 6.2142 (5) Å	θ = 2.3–27.5°
*b* = 21.1112 (13) Å	µ = 0.07 mm^−^^1^
*c* = 17.4624 (12) Å	*T* = 293 K
β = 92.869 (6)°	Block, colourless
*V* = 2288.0 (3) Å^3^	0.20 × 0.20 × 0.10 mm
*Z* = 4	

### Data collection

**Table:**

Rigaku SCXmini diffractometer	5239 independent reflections
Radiation source: fine-focus sealed tube	4315 reflections with *I* > 2σ(*I*)
graphite	*R*_int_ = 0.047
Detector resolution: 13.6612 pixels mm^-1^	θ_max_ = 27.5°, θ_min_ = 3.3°
ω scans	*h* = −8→8
Absorption correction: multi-scan (*CrystalClear*; Rigaku, 2005)	*k* = −27→27
*T*_min_ = 0.987, *T*_max_ = 0.993	*l* = −22→22
24819 measured reflections	

### Refinement

**Table:**

Refinement on *F*^2^	Primary atom site location: structure-invariant direct methods
Least-squares matrix: full	Secondary atom site location: difference Fourier map
*R*[*F*^2^ > 2σ(*F*^2^)] = 0.061	Hydrogen site location: inferred from neighbouring sites
*wR*(*F*^2^) = 0.135	H-atom parameters constrained
*S* = 1.09	*w* = 1/[σ^2^(*F*_o_^2^) + (0.0431*P*)^2^ + 0.936*P*] where *P* = (*F*_o_^2^ + 2*F*_c_^2^)/3
5239 reflections	(Δ/σ)_max_ < 0.001
302 parameters	Δρ_max_ = 0.22 e Å^−^^3^
0 restraints	Δρ_min_ = −0.18 e Å^−^^3^

### Special details

**Table:**

Geometry. All e.s.d.'s (except the e.s.d. in the dihedral angle between two l.s. planes) are estimated using the full covariance matrix. The cell e.s.d.'s are taken into account individually in the estimation of e.s.d.'s in distances, angles and torsion angles; correlations between e.s.d.'s in cell parameters are only used when they are defined by crystal symmetry. An approximate (isotropic) treatment of cell e.s.d.'s is used for estimating e.s.d.'s involving l.s. planes.
Refinement. Refinement of *F*^2^ against ALL reflections. The weighted *R*-factor *wR* and goodness of fit *S* are based on *F*^2^, conventional *R*-factors *R* are based on *F*, with *F* set to zero for negative *F*^2^. The threshold expression of *F*^2^ > σ(*F*^2^) is used only for calculating *R*-factors(gt) *etc*. and is not relevant to the choice of reflections for refinement. *R*-factors based on *F*^2^ are statistically about twice as large as those based on *F*, and *R*- factors based on ALL data will be even larger.

### Fractional atomic coordinates and isotropic or equivalent isotropic displacement parameters (Å^2^)

**Table:**

	*x*	*y*	*z*	*U*_iso_*/*U*_eq_	Occ. (<1)
N1	0.7010 (2)	0.17678 (6)	0.27399 (7)	0.0256 (3)	
N2	1.0343 (2)	0.14275 (6)	0.31033 (7)	0.0298 (3)	
C1	0.7112 (3)	0.17535 (7)	0.35346 (9)	0.0270 (3)	
C2	0.5570 (3)	0.18846 (8)	0.40650 (10)	0.0346 (4)	
H2B	0.4192	0.2021	0.3913	0.041*	
C3	0.6195 (3)	0.18016 (9)	0.48290 (10)	0.0392 (4)	
H3A	0.5214	0.1884	0.5201	0.047*	
C4	0.8268 (3)	0.15961 (9)	0.50544 (10)	0.0382 (4)	
H4A	0.8632	0.1547	0.5574	0.046*	
C5	0.9793 (3)	0.14633 (8)	0.45281 (10)	0.0344 (4)	
H5A	1.1170	0.1328	0.4684	0.041*	
C6	0.9185 (3)	0.15411 (7)	0.37498 (9)	0.0282 (3)	
C7	0.8992 (2)	0.15635 (7)	0.25137 (9)	0.0258 (3)	
C8	0.9583 (2)	0.14654 (7)	0.17169 (9)	0.0272 (3)	
C9	0.8876 (3)	0.18418 (8)	0.10959 (9)	0.0310 (4)	
H9A	0.7887	0.2164	0.1168	0.037*	
C10	0.9638 (3)	0.17391 (9)	0.03713 (10)	0.0355 (4)	
H10A	0.9141	0.1995	−0.0033	0.043*	
C11	1.1119 (3)	0.12648 (8)	0.02342 (10)	0.0344 (4)	
C12	1.1810 (3)	0.08896 (8)	0.08579 (10)	0.0370 (4)	
H12A	1.2802	0.0568	0.0785	0.044*	
C13	1.1055 (3)	0.09845 (8)	0.15806 (10)	0.0335 (4)	
H13A	1.1537	0.0723	0.1982	0.040*	
C14	1.1974 (3)	0.11519 (9)	−0.05650 (10)	0.0424 (5)	
C15	1.4372 (5)	0.1228 (2)	−0.0550 (2)	0.0569 (10)	0.636 (4)
H15A	1.4726	0.1670	−0.0576	0.085*	0.636 (4)
H15B	1.4930	0.1011	−0.0980	0.085*	0.636 (4)
H15C	1.4996	0.1051	−0.0083	0.085*	0.636 (4)
C16	1.1017 (6)	0.16598 (17)	−0.11722 (17)	0.0508 (9)	0.636 (4)
H16A	0.9475	0.1625	−0.1212	0.076*	0.636 (4)
H16B	1.1587	0.1580	−0.1664	0.076*	0.636 (4)
H16C	1.1414	0.2079	−0.1004	0.076*	0.636 (4)
C17	1.1189 (8)	0.0513 (2)	−0.0846 (2)	0.0687 (14)	0.636 (4)
H17A	0.9695	0.0541	−0.1011	0.103*	0.636 (4)
H17B	1.1360	0.0209	−0.0438	0.103*	0.636 (4)
H17C	1.2014	0.0381	−0.1268	0.103*	0.636 (4)
C16'	1.3128 (12)	0.1723 (4)	−0.0785 (4)	0.073 (2)	0.364 (4)
H16D	1.2128	0.2068	−0.0851	0.109*	0.364 (4)
H16E	1.3811	0.1648	−0.1258	0.109*	0.364 (4)
H16F	1.4201	0.1828	−0.0391	0.109*	0.364 (4)
C15'	1.3773 (13)	0.0576 (4)	−0.0524 (3)	0.077 (3)	0.364 (4)
H15D	1.3107	0.0193	−0.0360	0.116*	0.364 (4)
H15E	1.4934	0.0688	−0.0166	0.116*	0.364 (4)
H15F	1.4323	0.0514	−0.1023	0.116*	0.364 (4)
C17'	1.0230 (11)	0.0946 (4)	−0.1102 (3)	0.0593 (19)	0.364 (4)
H17D	0.9132	0.1266	−0.1137	0.089*	0.364 (4)
H17E	0.9627	0.0558	−0.0922	0.089*	0.364 (4)
H17F	1.0787	0.0879	−0.1598	0.089*	0.364 (4)
C18	0.5117 (2)	0.19700 (7)	0.22771 (9)	0.0276 (3)	
H18A	0.3834	0.1845	0.2533	0.033*	
H18B	0.5100	0.1753	0.1787	0.033*	
C19	0.5039 (2)	0.26793 (7)	0.21354 (8)	0.0244 (3)	
C20	0.6751 (3)	0.30771 (8)	0.23238 (10)	0.0345 (4)	
H20A	0.7992	0.2912	0.2568	0.041*	
C21	0.6647 (3)	0.37210 (8)	0.21545 (10)	0.0354 (4)	
H21A	0.7823	0.3976	0.2292	0.042*	
C22	0.4846 (2)	0.39941 (7)	0.17869 (9)	0.0255 (3)	
C23	0.3127 (3)	0.35908 (8)	0.16148 (11)	0.0380 (4)	
H23A	0.1875	0.3756	0.1379	0.046*	
C24	0.3214 (3)	0.29478 (8)	0.17827 (11)	0.0370 (4)	
H24A	0.2026	0.2694	0.1656	0.044*	
C25	0.4755 (3)	0.47028 (7)	0.15925 (10)	0.0314 (4)	
C26	0.6985 (3)	0.49533 (9)	0.14043 (12)	0.0454 (5)	
H26A	0.7489	0.4729	0.0970	0.068*	
H26B	0.7974	0.4891	0.1838	0.068*	
H26C	0.6887	0.5397	0.1287	0.068*	
C27	0.3234 (3)	0.48367 (9)	0.08955 (12)	0.0474 (5)	
H27A	0.3723	0.4612	0.0459	0.071*	
H27B	0.3218	0.5283	0.0791	0.071*	
H27C	0.1806	0.4699	0.1000	0.071*	
C28	0.3968 (4)	0.50619 (9)	0.22849 (12)	0.0586 (6)	
H28A	0.2570	0.4909	0.2404	0.088*	
H28B	0.3882	0.5506	0.2168	0.088*	
H28C	0.4959	0.4997	0.2717	0.088*	

### Atomic displacement parameters (Å^2^)

**Table:**

	*U*^11^	*U*^22^	*U*^33^	*U*^12^	*U*^13^	*U*^23^
N1	0.0275 (7)	0.0240 (6)	0.0255 (7)	0.0003 (5)	0.0032 (5)	0.0028 (5)
N2	0.0303 (7)	0.0312 (7)	0.0280 (7)	0.0019 (5)	0.0030 (6)	0.0021 (6)
C1	0.0328 (8)	0.0225 (7)	0.0259 (8)	−0.0023 (6)	0.0048 (6)	0.0025 (6)
C2	0.0335 (9)	0.0364 (9)	0.0344 (9)	0.0014 (7)	0.0075 (7)	0.0009 (7)
C3	0.0449 (11)	0.0425 (10)	0.0311 (9)	−0.0027 (8)	0.0118 (8)	−0.0010 (8)
C4	0.0482 (11)	0.0403 (10)	0.0263 (9)	−0.0046 (8)	0.0030 (7)	0.0035 (7)
C5	0.0375 (9)	0.0350 (9)	0.0306 (9)	−0.0009 (7)	0.0007 (7)	0.0044 (7)
C6	0.0321 (8)	0.0255 (8)	0.0271 (8)	−0.0018 (6)	0.0038 (6)	0.0023 (6)
C7	0.0281 (8)	0.0217 (7)	0.0277 (8)	−0.0012 (6)	0.0038 (6)	0.0016 (6)
C8	0.0281 (8)	0.0261 (8)	0.0276 (8)	−0.0049 (6)	0.0029 (6)	−0.0006 (6)
C9	0.0323 (9)	0.0304 (8)	0.0303 (9)	−0.0015 (7)	0.0032 (7)	0.0001 (7)
C10	0.0395 (10)	0.0388 (9)	0.0282 (9)	−0.0057 (7)	0.0023 (7)	0.0015 (7)
C11	0.0351 (9)	0.0387 (9)	0.0300 (9)	−0.0102 (7)	0.0067 (7)	−0.0082 (7)
C12	0.0371 (10)	0.0354 (9)	0.0391 (10)	0.0023 (7)	0.0076 (8)	−0.0077 (8)
C13	0.0353 (9)	0.0323 (9)	0.0330 (9)	0.0015 (7)	0.0034 (7)	−0.0003 (7)
C14	0.0468 (11)	0.0484 (11)	0.0333 (10)	−0.0098 (9)	0.0136 (8)	−0.0078 (8)
C15	0.048 (2)	0.078 (3)	0.047 (2)	0.0041 (18)	0.0199 (15)	0.0108 (18)
C16	0.056 (2)	0.070 (2)	0.0278 (15)	−0.0027 (17)	0.0097 (14)	0.0037 (15)
C17	0.105 (4)	0.059 (2)	0.045 (2)	−0.023 (2)	0.032 (2)	−0.0254 (19)
C16'	0.081 (6)	0.077 (5)	0.063 (4)	−0.019 (4)	0.041 (4)	−0.001 (4)
C15'	0.095 (6)	0.103 (6)	0.036 (3)	0.056 (5)	0.024 (3)	0.003 (4)
C17'	0.069 (4)	0.073 (5)	0.036 (3)	0.006 (3)	0.002 (3)	−0.015 (3)
C18	0.0254 (8)	0.0258 (8)	0.0316 (8)	−0.0026 (6)	−0.0001 (6)	0.0012 (6)
C19	0.0251 (8)	0.0247 (7)	0.0236 (7)	−0.0008 (6)	0.0039 (6)	−0.0004 (6)
C20	0.0289 (9)	0.0296 (8)	0.0437 (10)	−0.0010 (6)	−0.0107 (7)	0.0043 (7)
C21	0.0303 (9)	0.0272 (8)	0.0476 (11)	−0.0054 (6)	−0.0080 (7)	0.0018 (7)
C22	0.0273 (8)	0.0250 (7)	0.0243 (8)	0.0017 (6)	0.0041 (6)	−0.0007 (6)
C23	0.0271 (9)	0.0309 (9)	0.0548 (11)	0.0012 (7)	−0.0097 (8)	0.0059 (8)
C24	0.0260 (8)	0.0303 (9)	0.0538 (11)	−0.0057 (7)	−0.0074 (7)	0.0032 (8)
C25	0.0382 (9)	0.0248 (8)	0.0316 (9)	0.0022 (7)	0.0056 (7)	0.0022 (7)
C26	0.0454 (11)	0.0331 (9)	0.0576 (12)	−0.0072 (8)	0.0028 (9)	0.0099 (9)
C27	0.0487 (12)	0.0408 (10)	0.0525 (12)	0.0016 (9)	−0.0011 (9)	0.0176 (9)
C28	0.1005 (19)	0.0285 (9)	0.0493 (12)	0.0113 (10)	0.0287 (12)	0.0019 (9)

### Geometric parameters (Å, °)

**Table:**

N1—C7	1.3816 (19)	C17—H17A	0.9600
N1—C1	1.386 (2)	C17—H17B	0.9600
N1—C18	1.4571 (19)	C17—H17C	0.9600
N2—C7	1.327 (2)	C16'—H16D	0.9600
N2—C6	1.390 (2)	C16'—H16E	0.9600
C1—C2	1.393 (2)	C16'—H16F	0.9600
C1—C6	1.398 (2)	C15'—H15D	0.9600
C2—C3	1.382 (2)	C15'—H15E	0.9600
C2—H2B	0.9300	C15'—H15F	0.9600
C3—C4	1.397 (3)	C17'—H17D	0.9600
C3—H3A	0.9300	C17'—H17E	0.9600
C4—C5	1.381 (2)	C17'—H17F	0.9600
C4—H4A	0.9300	C18—C19	1.518 (2)
C5—C6	1.402 (2)	C18—H18A	0.9700
C5—H5A	0.9300	C18—H18B	0.9700
C7—C8	1.471 (2)	C19—C20	1.382 (2)
C8—C13	1.395 (2)	C19—C24	1.385 (2)
C8—C9	1.397 (2)	C20—C21	1.392 (2)
C9—C10	1.390 (2)	C20—H20A	0.9300
C9—H9A	0.9300	C21—C22	1.387 (2)
C10—C11	1.389 (2)	C21—H21A	0.9300
C10—H10A	0.9300	C22—C23	1.387 (2)
C11—C12	1.397 (3)	C22—C25	1.535 (2)
C11—C14	1.536 (2)	C23—C24	1.389 (2)
C12—C13	1.383 (2)	C23—H23A	0.9300
C12—H12A	0.9300	C24—H24A	0.9300
C13—H13A	0.9300	C25—C28	1.528 (2)
C14—C17'	1.463 (6)	C25—C27	1.530 (2)
C14—C16'	1.465 (7)	C25—C26	1.534 (2)
C14—C15	1.498 (4)	C26—H26A	0.9600
C14—C17	1.508 (4)	C26—H26B	0.9600
C14—C16	1.601 (4)	C26—H26C	0.9600
C14—C15'	1.650 (6)	C27—H27A	0.9600
C15—H15A	0.9600	C27—H27B	0.9600
C15—H15B	0.9600	C27—H27C	0.9600
C15—H15C	0.9600	C28—H28A	0.9600
C16—H16A	0.9600	C28—H28B	0.9600
C16—H16B	0.9600	C28—H28C	0.9600
C16—H16C	0.9600		
			
C7—N1—C1	106.40 (13)	C14—C17—H17A	109.5
C7—N1—C18	129.74 (13)	C14—C17—H17B	109.5
C1—N1—C18	123.86 (13)	H17A—C17—H17B	109.5
C7—N2—C6	105.05 (13)	C14—C17—H17C	109.5
N1—C1—C2	131.48 (15)	H17A—C17—H17C	109.5
N1—C1—C6	105.77 (13)	H17B—C17—H17C	109.5
C2—C1—C6	122.70 (15)	C14—C16'—H16D	109.5
C3—C2—C1	116.56 (16)	C14—C16'—H16E	109.5
C3—C2—H2B	121.7	H16D—C16'—H16E	109.5
C1—C2—H2B	121.7	C14—C16'—H16F	109.5
C2—C3—C4	121.47 (16)	H16D—C16'—H16F	109.5
C2—C3—H3A	119.3	H16E—C16'—H16F	109.5
C4—C3—H3A	119.3	C14—C15'—H15D	109.5
C5—C4—C3	121.94 (16)	C14—C15'—H15E	109.5
C5—C4—H4A	119.0	H15D—C15'—H15E	109.5
C3—C4—H4A	119.0	C14—C15'—H15F	109.5
C4—C5—C6	117.44 (16)	H15D—C15'—H15F	109.5
C4—C5—H5A	121.3	H15E—C15'—H15F	109.5
C6—C5—H5A	121.3	C14—C17'—H17D	109.5
N2—C6—C1	110.16 (13)	C14—C17'—H17E	109.5
N2—C6—C5	129.95 (15)	H17D—C17'—H17E	109.5
C1—C6—C5	119.88 (15)	C14—C17'—H17F	109.5
N2—C7—N1	112.61 (14)	H17D—C17'—H17F	109.5
N2—C7—C8	121.68 (14)	H17E—C17'—H17F	109.5
N1—C7—C8	125.63 (14)	N1—C18—C19	113.43 (12)
C13—C8—C9	117.69 (15)	N1—C18—H18A	108.9
C13—C8—C7	117.42 (14)	C19—C18—H18A	108.9
C9—C8—C7	124.78 (14)	N1—C18—H18B	108.9
C10—C9—C8	120.65 (16)	C19—C18—H18B	108.9
C10—C9—H9A	119.7	H18A—C18—H18B	107.7
C8—C9—H9A	119.7	C20—C19—C24	117.44 (14)
C11—C10—C9	121.88 (16)	C20—C19—C18	122.86 (14)
C11—C10—H10A	119.1	C24—C19—C18	119.67 (14)
C9—C10—H10A	119.1	C19—C20—C21	121.05 (15)
C10—C11—C12	117.05 (15)	C19—C20—H20A	119.5
C10—C11—C14	122.04 (17)	C21—C20—H20A	119.5
C12—C11—C14	120.91 (16)	C22—C21—C20	122.13 (15)
C13—C12—C11	121.63 (16)	C22—C21—H21A	118.9
C13—C12—H12A	119.2	C20—C21—H21A	118.9
C11—C12—H12A	119.2	C23—C22—C21	116.09 (14)
C12—C13—C8	121.10 (16)	C23—C22—C25	122.07 (14)
C12—C13—H13A	119.5	C21—C22—C25	121.83 (14)
C8—C13—H13A	119.5	C22—C23—C24	122.19 (15)
C17'—C14—C16'	115.7 (5)	C22—C23—H23A	118.9
C17'—C14—C15	138.4 (3)	C24—C23—H23A	118.9
C16'—C14—C15	54.3 (4)	C19—C24—C23	121.07 (15)
C17'—C14—C17	46.3 (3)	C19—C24—H24A	119.5
C16'—C14—C17	143.7 (3)	C23—C24—H24A	119.5
C15—C14—C17	114.0 (3)	C28—C25—C27	109.18 (16)
C17'—C14—C11	110.5 (3)	C28—C25—C26	109.07 (16)
C16'—C14—C11	107.9 (3)	C27—C25—C26	107.14 (15)
C15—C14—C11	110.84 (19)	C28—C25—C22	108.57 (14)
C17—C14—C11	108.26 (19)	C27—C25—C22	111.76 (14)
C17'—C14—C16	62.4 (3)	C26—C25—C22	111.08 (14)
C16'—C14—C16	56.3 (4)	C25—C26—H26A	109.5
C15—C14—C16	106.1 (2)	C25—C26—H26B	109.5
C17—C14—C16	106.3 (3)	H26A—C26—H26B	109.5
C11—C14—C16	111.34 (18)	C25—C26—H26C	109.5
C17'—C14—C15'	106.7 (4)	H26A—C26—H26C	109.5
C16'—C14—C15'	106.2 (5)	H26B—C26—H26C	109.5
C15—C14—C15'	53.6 (4)	C25—C27—H27A	109.5
C17—C14—C15'	64.1 (4)	C25—C27—H27B	109.5
C11—C14—C15'	109.8 (2)	H27A—C27—H27B	109.5
C16—C14—C15'	138.6 (3)	C25—C27—H27C	109.5
C14—C15—H15A	109.5	H27A—C27—H27C	109.5
C14—C15—H15B	109.5	H27B—C27—H27C	109.5
H15A—C15—H15B	109.5	C25—C28—H28A	109.5
C14—C15—H15C	109.5	C25—C28—H28B	109.5
H15A—C15—H15C	109.5	H28A—C28—H28B	109.5
H15B—C15—H15C	109.5	C25—C28—H28C	109.5
C14—C16—H16A	109.5	H28A—C28—H28C	109.5
C14—C16—H16B	109.5	H28B—C28—H28C	109.5
C14—C16—H16C	109.5		

### Hydrogen-bond geometry (Å, °)

**Table:**

*D*—H···*A*	*D*—H	H···*A*	*D*···*A*	*D*—H···*A*
C18—H18A···N2^i^	0.97	2.59	3.553 (2)	174

Symmetry codes: (i) *x*−1, *y*, *z*.
